# A novel combination of sodium metabisulfite and a chemical mixture based on sodium benzoate, potassium sorbate, and sodium nitrite for aerobic preservation of fruit and vegetable discards and lactic acid fermentation in a total mixed ration for ruminants

**DOI:** 10.5713/ab.20.0871

**Published:** 2021-03-02

**Authors:** Farhad Ahmadi, Won Hee Lee, Wan Sup Kwak

**Affiliations:** 1 Food Bio-science Major, College of Medical Life Sciences, Konkuk University, Chungju 27478, Korea

**Keywords:** Anaerobic Fermentation, Fruit and Vegetable Waste, *Lactobacillus plantarum*, Ruminant, Total Mixed Ration

## Abstract

**Objective:**

Our recent findings confirmed the effectiveness of sodium metabisulfite (SMB) in controlling the growth of undesirable microorganisms in fruit and vegetable discards (FVD); however, lactic acid bacteria (LAB) are susceptible to its antibacterial effects. Two series of experiments were conducted to enable the survivability of LAB during silage fermentation of a total mixed ration (TMR) containing SMB-treated FVD.

**Methods:**

In Exp. 1, the objective was to isolate a strain of LAB tolerable to the toxic effect of SMB. In Exp. 2, the SMB load was minimized through its partial replacement with a chemical mixture (CM) based on sodium benzoate (57%), potassium sorbate (29%), and sodium nitrite (14%). FVD was treated with SMB + CM (2 g each/kg biomass) and added to the TMR at varying levels (0%, 10%, or 20%), with or without KU18 inoculation.

**Results:**

The KU18 was screened as a presumptive LAB strain showing superior tolerance to SMB in broth medium, and was identified at the molecular level using 16S rRNA gene sequence analysis as *Lactobacillus plantarum*. Inoculation of KU18 in TMR containing SMB was not successful for the LAB development, biomass acidification, and organoleptic properties of the resultant silage. In Exp. 2, based on the effectiveness and economic considerations, an equal proportion of SMB and CM (2 g each/kg FVD) was selected as the optimal loads for the subsequent silage fermentation experiment. Slight differences were determined in LAB development, biomass acidification, and sensorial characteristics among the experimental silages, suggesting the low toxicity of the preservatives on LAB growth.

**Conclusion:**

Although KU18 strain was not able to efficiently develop in silage mass containing SMB-treated FVD, the partial substitution of SMB load with the CM effectively alleviated the toxic effect of SMB and allowed LAB development during the fermentation of SMB + CM-treated FVD in TMR.

## INTRODUCTION

Primary observations from our research team showed that fruit and vegetable discards (FVD) are a rich source of soluble sugars with high water content that jointly provide a suitable medium for the growth and proliferation of undesirable microorganisms responsible for fast deterioration of FVD biomass [1–3]. This problem directed our focus to find a proper preservative for the effective storage and safe usage of FVD biomass as a source of animal feed. We selected sodium metabisulfite (SMB) because it is inexpensive, readily available, and its handling is easy and safe [2,4]. Our recent series of laboratory- and large-scale experiments confirmed that SMB applied at the rate of 6 g/kg wet biomass was the minimum load able to provide strong inhibition against a wide range of microorganisms that enabled the effective preservation of FVD under both aerobic and anaerobic storage [1–3].

The high moisture content of FVD presents hurdles associated with their transportation and handling [1]. One of the easiest and most efficient methods is mixing them with dry feeds and storage through ensiling which would i) avoid the high energy costs associated with drying, ii) minimize the effluent production, and iii) help to improve the preservation stability and nutritional value of FVD for use as animal feed [1,5,6]. In ruminant production systems, there is a growing interest among total mixed ration (TMR) manufacturers to produce TMR in the form of transportable bag silos (300 to 400 kg capacity) which enable the effective anaerobic fermentation and thus small nutrient loss during storage. This practice provides labor savings, the opportunity to include perishable high-moisture byproducts, in addition to some nutrient transformations during the ensiling process, which improve the feeding value of TMR silage for ruminants [6,7]. Importantly, lactic acid fermentation of TMR ingredients could help masking the flavor of unpalatable ingredients such as FVD, which is important as this approach minimizes the off-feed problems with the addition of FVD in TMR, and thus improves their odor and flavor through fermentation during ensiling [5,6]. One of the most important factors in producing high-quality silage is the fast and effective development of lactic acid bacteria (LAB) in the silage microbial community as the fermentation proceeds [8]. However, our findings showed that among the microorganisms studied, LAB were the most sensitive to the bactericidal effect of SMB [1–3]. Irwin et al [9] studied the susceptibility and resistance of LAB to sodium bisulfite in De Man, Rogosa and Sharpe (MRS) broth and reported a half maximal inhibitory concentration of 436 mg/L for *Lactobacillus plantarum* (*L. plantarum*).

Recent studies suggested that a water solution of sodium benzoate (200 g/L), potassium sorbate (100 g/L), and sodium nitrite (50 g/L) at an application rate of 5 mL of mixture per kg fresh biomass improved the silage fermentation quality and prolonged the aerobic stability of various crop materials, which was largely related to the suppression of the growth of undesirable microflora [10–12]. Knicky and Spörndly [10,11] added the same chemical mixture (CM) based on sodium benzoate, potassium sorbate, and sodium nitrite at a rate of 5 mL/kg of fresh crop to a number of crop materials. Interestingly, these authors reported that the CM treatment resulted in substantially lower pH values and increased lactic acid concentration at the end of the silage fermentation compared to the control, which indicates the dominance of LAB in the silage fermentation process.

Two series of experiments were conducted to minimize the inhibitory effects of SMB on LAB survivability and growth. The first approach was to test the possibility of isolating a strain of a lactic acid bacterium resistant to SMB that would be hypothetically able to grow under the anaerobic environment of SMB-containing silage biomass. The second approach was to minimize the load of SMB with its partial replacement with the CM that does not have inhibitory effect on growth and development of LAB. We hypothesized that minimizing the SMB load would result in a lesser inhibitory effect on LAB, thus allowing their growth and development in silage mass.

## MATERIALS AND METHODS

### Fruit and vegetable discards preparation and lactic acid bacteria isolation (Exp. 1)

Five different batches of FVD were obtained in different days from a commercial packinghouse (E-mart Fresh Logistic Center, Icheon, Korea), homogenized until uniform size, and then anaerobically fermented in 5 L glass jars for 14 days at room temperature (25°C). After silo opening, the representative FVD samples (10 g) were obtained from the center of the silage mass, blended with 90 mL of sterilized distilled water, and serially diluted at 10^−3^ to 10^−8^ with sterilized distilled water. The 100-μL aliquot of the serially-diluted slurry was inoculated using the spread-plating method on MRS agar (Difco Laboratories, Detroit, MI, USA). A total of 42 presumptive LAB colonies differing in morphological characteristics (colony size, shape, and color) were selected after incubation at 30°C for 48 h under anaerobic conditions. The morphological characterization was based on Bergey’s Manual of Systematic Bacteriology [13]. Each single presumptive LAB colony was purified twice by streaking onto MRS agar. The pure cultures were grown on MRS agar (30°C for 24 h) and given a temporary number. The cultures were then stored in MRS broth plus 20% (v/v) sterile glycerol as stock cultures at −80°C [13].

The presumptive LAB isolates were reactivated and sub-cultured in MRS broth (30°C; for 18 to 20 h) and then inoculated (1% v/v) under aseptic conditions into 50 mL of fresh MRS broth. MRS broth media were prepared freshly according to the manufacturer’s instructions. The isolates that yielded cell counts higher than 2×10^9^/mL of culture [14] (data not presented) were selected (29 isolates) for their further screening using a test to identify the LAB strain most tolerant to SMB. Each of the viable LAB isolates was cultured in triplicates in MRS broth (200 mL) and SMB (1,000 mg/L) was added. SMB (Sigma Chemical Co.; Saint Louis, MO, USA) was dissolved in sterile water (20% w/v) and filter-sterilized through a 0.45 μm pore size filter and then added to MRS media before use. The cultures were incubated at 30°C for 24 h, plated on MRS agar plates using the spread-plating method, and viable LAB colonies were enumerated after incubation. Five strains exhibiting the highest growth potential were selected for subsequent examination in SMB-added MRS broth to check for changes in culture pH as a function of the incubation time (3, 6, 12, and 24 h). *L. plantarum* KU5 (accession No. HQ542227) was used as a positive control strain [15]. Finally, from a rank assessment based on viable LAB counts and growth rate, one potential LAB candidate was selected for the molecular identification using the 16S ribosomal RNA sequencing analysis. The sequencing procedure was performed in a commercial laboratory (Korea Research Institute of Bio-medical Science, Korea). For DNA extraction and purification, LAB cells were grown on MRS broth at 30°C for 8 h. DNA was further extracted using a commercially available kit (Bioneer Co., Daejon, Korea) as per the manufacturer’s instructions. For the amplification of 16S rRNA genes, the 27F and 1492R universal primers were used and their sequence is as follows: 27F (5′-AGAG TTTGATCMTGGCTCAG-3′) and 1492R (5′-TACGGYT ACCTTGTTACGACT-3′). The 16S rRNA sequence similarity searches were obtained from the GenBank data library with the BLAST algorithm program. For assembly and alignment, the sequence information was imported into the Clustal X 1.81 software program. The KU18 sequence was compared to sequences from LAB-type strains held in GenBank. After calculation of nucleotide substitution rates, a phylogenetic tree was constructed using the neighbor-joining method [16].

For the silage fermentation experiment, the representative FVD samples were obtained, ground to uniform size using a meat grinder, and mixed with 6 g SMB/kg fresh FVD mass [1]. The individual ingredient proportion and the chemical composition of FVD are presented in Supplementary Table S1. After 7 days of aerobic exposure, SMB-treated FVD was included in the TMR at 0%, 10%, or 20% level on an as-fed basis (corresponding to 0%, 25%, and 50% of wet brewers’ grain replaced by SMB-treated FVD). The maximum level inclusion in TMR was set at 20% (as-fed basis) because of the high moisture content of FVD (85% to 88%) [1,2]. Before packing into the silos, the TMR ingredients were thoroughly mixed for 10 min using an electric mixer (Akita, Verona, Italy). For the inoculated treatment, the KU18 strain was sprayed at the rate of 5 mL/kg biomass, supplying 1×10^6^ colony-forming unit (cfu)/g of fresh biomass. The TMR mass (20 kg) was packed into polyvinyl bags (70 cm wide× 100 cm long), and allowed to ferment at room temperature (19°C to 25°C) for 14 days. A non-inoculated treatment was included as a negative control.

### Combination of sodium metabisulfite and chemical mixture as preservative (Exp. 2)

In this experiment, SMB was replaced at varying ratios with a CM based on sodium benzoate (57%), potassium sorbate (29%), and sodium nitrite (14%). Treatments (g preservative per kg biomass) were: i) control (no preservative); ii) 6 g SMB; iii) 3 g CM; iv) SMB + CM in equal amounts (2 g each); v) 3 g SMB + 1.5 g CM; and vi) 4 g SMB + 1 g CM. FVD was ground to uniform size as indicated in Exp. 1, mixed with varying ratios of SMB to CM, and exposed to the 7-day aerobic challenge. For the silage fermentation experiment, groups of five samples were allocated to one of the following six treatments: i) control TMR with no KU18 inoculant; ii) control TMR with KU18 inoculant; iii) TMR with 10% FVD (mixed with 2 g CM + 2 g SMB/kg fresh biomass); iv) TMR with 10% FVD (mixed with 2 g CM + 2 g SMB/kg fresh biomass) + KU18 inoculant; v) TMR with 20% FVD (mixed with 2 g CM + 2 g SMB/kg fresh biomass); and vi) TMR with 20% FVD (mixed with 2 g CM + 2 g SMB/kg fresh biomass) + KU18 inoculant. Before ensiling, the KU18 inoculant was recultivated in MRS broth (24 h at 30°C) and the properly diluted suspension was uniformly sprayed onto the TMR mass at a rate of 5 mL/kg biomass to supply 1×10^6^ cfu/g of fresh biomass. The TMR ingredients were thoroughly mixed with the mixer for 10 min, packed into polyvinyl bags (20 kg each), and allowed to ferment at room temperature for 14 days. In both experiments a packing density of about 200 kg dry matter (DM)/m^3^ was applied to all silages.

### Analytical procedures

To quantify the number of viable colonies in FVD biomass and silage mass, a water extract was prepared using the method of Ahmadi et al [2]. In brief, a 20-g sample of fresh or ensiled biomass was mixed with 120 mL of distilled sterile water. The slurry was shaken for 5 min and filtered through a 2-layered cheese gauze. The slurry was serially diluted, plated on yeast extract glucose chloramphenicol agar, incubated at 25°C for 3 days for yeast and 5 days for mold [1], and colonies enumerated. The pH was determined using a pH meter (HI 9321, Hanna Instruments, Woonsocket, RI, USA). Another portion of the slurry was centrifuged at 10,000 *g* for 10 min and the supernatant collected for quantification of water-soluble carbohydrates (WSC) using the method of Dubois et al [17]. Crude protein (N×6.25), crude ash, DM, ether extract, and neutral-detergent fiber (NDF) contents were determined using the methods of AOAC [18]. Non-fibrous carbohydrates (NFC) were calculated as 100–[%NDF + % crude protein + % crude ash + % ether extract].

### Statistical analysis

For the aerobic preservation study, the treatment difference (effect of preservation day) was identified using a *t*-test at the 0.05 probability level. Data were analyzed using the Proc Mixed of SAS (version 9.1; SAS Institute, Inc., Cary, NC, USA). The model used for the analysis was: Y_ij_ = μ+T_i_+e_ij_, where Y_ij_ = measured value for each observation, μ = mean, T_i_ = fixed effect of treatment, and e_ij_ = the residual error. Replicates were considered as a random effect in the model. The difference between treatments was identified using the Tukey’s multiple test range at the 0.05 probability level. The replicate bucket or polybag silo served as the experimental unit.

## RESULTS AND DISCUSSION

### Isolation of sodium metabisulfite-tolerant lactic acid bacteria (Exp. 1)

This experiment aimed to screen a selection of the best performing LAB isolates for their ability to grow in the presence of SMB and then confirm their potential as silage inoculants in laboratory-scale silos. LAB are very vulnerable to the toxic effects of SMB [19]. Sulfiting agents such as SMB are known to inactivate vital enzymes involved in nicotinamide adenine dinucleotide and ATP production, thereby resulting in microbial cell death [20,21]. The high susceptibility of LAB to the toxic effects of SMB might be explained by the formation of sulfur trioxide anion radicals, which are highly toxic and generated by the reaction of sulfite with hydrogen peroxide, which is produced in variable amounts by LAB under different environmental conditions. Therefore, the generation of H_2_O_2_ in the presence of LAB might explain the intensification of the toxic effects of SMB on cell death [9,22].

**Figure 1:**
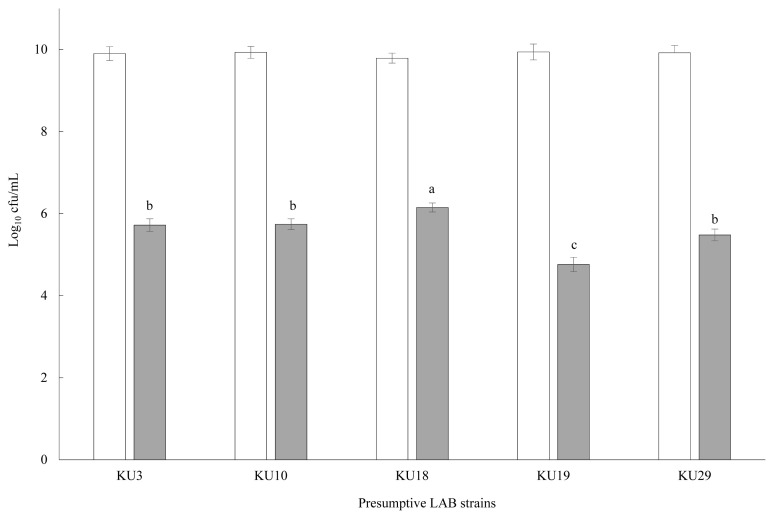
Viable LAB count of five isolated LAB in MRS broth with (grey bar) or without (open bar) sodium metabisulfite addition (1,000 mg/L) after 24 h of incubation. Error bars at each point represent standard error (n = 5). LAB, lactic acid bacteria; MRS, De Man, Rogosa and Sharpe. Bars with different letters differ, based on the Tukey’s test (p = 0.04).

The enumeration (log_10_ cfu/mL) of the 29 preliminary selected LAB isolates incubated for 12 h in MRS broth with SMB addition (1,000 mg/L) is shown in Table 1. Four presumptive LAB strains failed to remain viable in SMB-containing MRS broth and their count declined to below detectable levels. Comparatively, LAB counts of KU3 (5.98), KU10 (5.83), KU18 (6.10), KU19 (5.18), and KU29 (5.71) were higher than for the other strains and thus, selected for further examination. LAB counts of the five preliminary selected LAB (KU3, KU10, KU18, KU19, and KU29) incubated 24 h in MRS broth with or with no SMB addition (1,000 mg/L) is shown in Figure 1. Viable LAB colonies in MRS broth with no SMB addition ranged from 9.94 in KU19 to 9.90 log_10_ cfu/mL in KU3. However, SMB addition resulted in a substantial reduction in their count, ranging from 6.39 in KU18 to 4.70 log_10_ cfu/mL in KU19. Although the graph clearly shows the strong suppressing effect of SMB on LAB growth, KU18 showed a comparatively higher growth potential than the other strains.

The culture pH of the 5 selected LAB in MRS broth with SMB addition as a function of the incubation time (0, 3, 6, 12, and 24 h) is shown in Table 2. A slight difference was observed in the pH within 3 h of incubation, thereafter the rate of the pH reduction was faster in the KU18 strain compared to the other isolated strains and the *L. plantarum* KU5 positive control, leading to the lowest value of 5.71 after 24 h of incubation (p<0.01). Based on the growth potential and pH reduction in SMB-added MRS broth, KU18 was considered as the most SMB-tolerant LAB strain since it was able to comparatively grow more than the other strains and thus, was selected for further identification at the molecular level using 16S rRNA sequencing analysis. The 16S rRNA sequence description of KU18 is shown in Supplementary Figure S1. The phylogenetic tree by 16S rRNA sequencing analysis showed that KU18 had 99.0% similarity to *L. plantarum* (99%) in the GenBank database.

In the next phase, the KU18 strain was used as a silage inoculant for additional investigations in silage fermentation experiments. The 7-days aerobically exposed FVD preserved with SMB were included in a TMR in order to confirm the efficacy of KU18 in dominating the silage microbial community. A negligible difference was determined in the chemical composition of SMB-treated FVD before and after 7 day of aerobic storage (data not presented) in agreement with our previous findings that revealed the effectiveness of SMB in preserving the nutrient constituents of FVD under aerobic conditions [1–3,23]. The 7-days aerobic exposure was selected to simulate the practical situation of FVD amassment in the source of generation as revealed by our previous on-site survey [2].

FVD was selected as a replacement to wet brewers’ grain in TMR because of their similarity of DM and energy content. The ingredient and chemical composition of the TMR is presented in Supplementary Table S2. The DM, crude protein, and NDF contents of the TMR averaged 63.6%, 12.9% of DM, and 36.9% of DM, respectively. With increasing SMB-treated FVD level in TMR, the crude protein and DM contents slightly decreased, which could be explained by the lower crude protein and DM content of FVD than in wet barley brewers’ grain. NFC and WSC contents increased slightly as the level of SMB-treated FVD increased in TMR because of the higher concentrations of NFC (72.7% of DM) and WSC (43.6% of DM) in FVD than in wet brewers’ grain.

**Figure 2:**
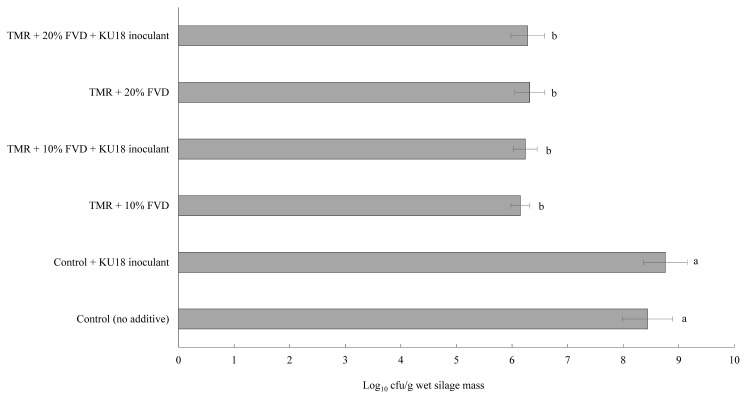
Viable LAB count of TMR silage after 14 days of fermentation with different levels of FVD. FVD were mixed with 6 g SMB/kg fresh biomass, and aerobically exposed for 7 days at an outdoor environment. Treatments were TMR containing (as-fed basis) 0%, 10%, or 20% SMB-treated FVD, with or without KU18 inoculant. The inoculant was applied uniformly at the rate of 5 mL/kg fresh mass (Exp. 1). Error bars at each point represent standard error (n = 5). LAB, lactic acid bacteria; TMR, total mixed ration; FVD, fruit and vegetable discards; SMB, sodium metabisulfite. Bars with different letters differ, based on the Tukey’s test (p<0.01).

Viable LAB colony count of TMR silage after 14 days of fermentation at different FVD levels and treated with 6 g SMB/kg fresh FVD mass, with or with no KU18 inoculation, is shown in Figure 2. Little variation was determined in the initial LAB population among the KU18-inoculated silages with or without SMB-treated FVD, averaging 7.78 log_10_ cfu/g of biomass (data not presented), which is higher than the minimum adequate number of 5 log_10_ cfu/g known to promote an efficient silage fermentation [24]. This also suggests that the inclusion of SMB-treated FVD in TMR had no immediate suppressive effect on LAB viability. As expected, in the control TMR with no SMB-treated FVD, the LAB count reached high numbers of 8.44 and 8.76 log_10_ cfu/g fresh silage mass after 14 days of fermentation, in non-inoculated and KU18-inoculated silages, respectively. However, a small difference was observed in the LAB number of silages containing 10% or 20% SMB-treated FVD with or with no KU18 inoculant, averaging 6.25 log_10_ cfu/g fresh silage mass after 14 days of ensiling. The progressive decline of the LAB count in TMR silage containing SMB-treated FVD clearly shows the inability of LAB (epiphytes or KU18) to develop and dominate the silage microbial community during silage fermentation.

The sensorial evaluation of the TMR silages also revealed that although the control TMR silage either with or with no KU18 inoculation, had desirable organoleptic properties which are indicative of successful lactic acid fermentation, a putrid odor was perceived in TMR silages containing SMB-treated FVD, especially at the 20% level, and regardless of KU18 inoculation. This observation may suggest inefficient fermentation due primarily to an insufficient LAB population inhibited by SMB. The adequate number of LAB enables the considerable production of lactic acid, which is known to suppress the growth of undesirable microorganisms responsible for putrefaction such as enterobacteria, clostridia, and yeasts, and thus result in silage with favorable fermentative smell [8].

**Figure 3:**
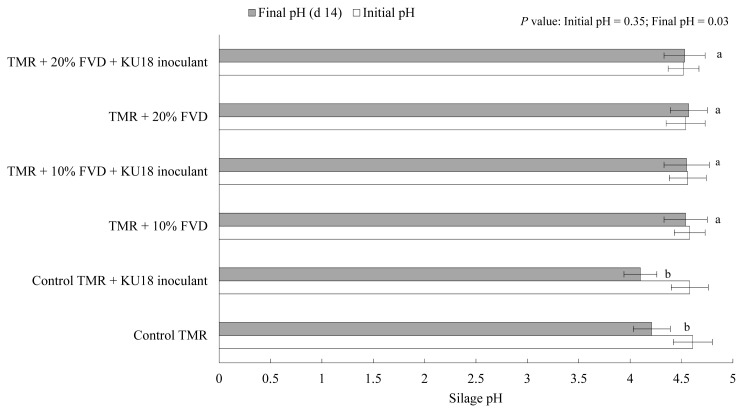
Initial (open bar) and final pH (grey bar; day 14) of TMR silage with different levels of FVD. FVD were mixed with 6 g SMB/kg fresh biomass, and aerobically exposed for 7 days at an outdoor environment. Treatments were TMR containing (as-fed basis) 0%, 10%, or 20% SMB-treated FVD, with or without KU18 inoculant. The inoculant was applied uniformly at the rate of 5 mL/kg fresh mass. Error bars at each point represent standard error (n = 5). TMR, total mixed ration; FVD, fruit and vegetable discards; SMB, sodium metabisulfite. Bars (grey bar) with different letters differ, based on the Tukey’s test.

The initial and final pH of TMR silage at different levels of SMB-treated FVD, with or with no KU18 inoculation, is shown in Figure 3. Although a narrow difference was observed in the initial pH of samples at day 0, the pH slightly declined with the increase of the level of SMB-treated FVD in TMR, the average of 4.57 among experimental treatments conducted, which is related to the acidic nature of SMB-treated FVD [1,2]. The lowest numerical pH value was determined in the control TMR silage inoculated with KU18, which is likely suggestive of the success of KU18 in promoting the acidification of silage mass, evidenced by the relatively higher count of viable LAB in the silage treatment. However, a slight difference was determined in the initial and final pH of silages containing SMB-treated FVD (10% or 20%) regardless of the KU18 inoculation. This might be explained by the toxic effect of SMB that inhibited the growth and development of the LAB population during the silage fermentation process (Figure 2). Overall, the KU18 strain showed a stronger growth potential in SMB-added MRS broth medium compared to the other screened strains; however, the strain was not able to efficiently survive and dominate in TMR silage containing SMB-treated FVD and provide the silage with desirable organoleptic properties.

### Combination of sodium metabisulfite and chemical mixture as preservative (Exp. 2)

The first objective of this experiment was to minimize the load of SMB through its replacement with a CM based on sodium benzoate, potassium sorbate, and sodium nitrite, which was reported as having no inhibitory effect on LAB growth and lactic acid production during silage fermentation [11]. The market price of SMB is much lower than CM ($261/MT versus $1,897/MT; Echemi Market Analysis, 2021; www.Enchemi.com); therefore, the minimization of CM load through its replacement with SMB will improve the cost-effectiveness of the preservation process. The second objective was to investigate if SMB-tolerant LAB (KU18) might act as a silage inoculant when the SMB load decreased. Although the antimicrobial properties of sodium benzoate, potassium sorbate, sodium nitrite, and their varying combinations have already been reported [25,26], to our knowledge, their efficacy on FVD biomass under aerobic and anaerobic storage has not been reported. Combination of sodium benzoate (200 g/L), potassium sorbate (100 g/L), and sodium nitrite (50 g/L) was demonstrated to improve the silage quality of both high- and low-DM silages [12]. This mixture was applied at the rate of 5 mL/kg of fresh crop (red clover, timothy and meadow fescue, or whole maize crop) and resulted in the reduction of undesirable microorganisms such as yeast and mold and the increase of lactic acid production during silage fermentation. These two effects prolonged the aerobic stability even under air-challenged ensiling conditions [11]. These results led us to hypothesize that the combination might be an effective preservative for high-moisture FVD.

The treatment effects on the microbial population and the pH of FVD before and after the 7-days aerobic challenge are presented in Table 3. The initial numbers of yeast, mold, LAB, and total bacteria declined considerably in FVD treated with SMB, CM, or their combinations compared to the control. For example, the number of yeast and mold was below the detection limit (2.8 log cfu per g fresh matter) in FVD treated with SMB or CM, which highlights the immediate inhibitory effect of SMB or CM on these microorganisms. After 7 days of aerobic storage, there was a substantial increase in the viable colony number of total bacteria (8.26 log_10_ cfu/g biomass) and LAB (7.26 log_10_ cfu/g biomass) in the control FVD. The viable numbers of LAB, yeast, and mold decreased to levels below the limit of detection in FVD treated with SMB, CM, or their combination, with the exception of CM-only treatment that had a LAB count of 3.11 log_10_ cfu/g biomass.

A substantial pH decline was determined in FVD with no preservative after 7 days of aerobiosis, which was expected as the number of total bacteria markedly increased and WSC declined considerably. This observation also agrees with our previous report that found a 0.66-unit pH reduction in FVD with no preservative after 9 days of aerobic exposure [2]. It was suggested that when the acid production rate surpasses the degradation rate, a pH drop occurs [2,27]. The pH change was narrower in FVD treated with an equal combination of SMB and CM, but declined to a greater extent when FVD was treated with SMB (3 g) + CM (1.5 g).

**Figure 4:**
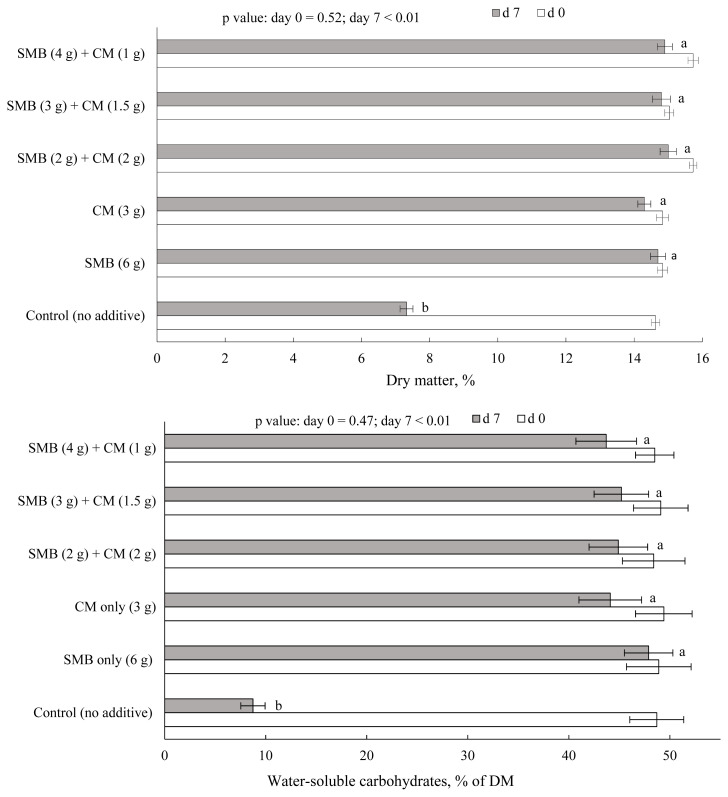
Dry matter and water-soluble carbohydrates content of fruit and vegetable discards at 0 (open bar) or 7 days (grey bar) of aerobic exposure. Treatments (g preservative/kg biomass) were i) control (no preservative); ii) 6 g sodium metabisulfite (SMB); iii) 3 g chemical mixture (CM); iv) 2 g SMB+2 g CM; v) 3 g SMB+1.5 g CM; and vi) 4 g SMB+1 g CM. CM was based on sodium benzoate (57%), potassium sorbate (29%), and sodium nitrite (14%). Bars with different letters differ, based on the Tukey’s test. Error bars at each point represent standard error (n = 5).

The effect of the treatment on DM and WSC contents of FVD before and after 7 days of aerobic exposure is shown in Figure 4. A substantial loss of DM and WSC was observed in FVD mass with no preservative (control). This result was expected as the total number of bacteria, yeast, and mold increased noticeably in FVD after aerobic storage, and microorganisms quickly exhaust the soluble sugars and convert them to water and carbon dioxide [1,2,28]. Loss of DM and WSC contents were negligible in FVD treated with SMB, CM, or their combinations. For example, only 7% of the WSC were lost in FVD treated with the equal ratio of SMB and CM during the 7 days of aerobic exposure. These findings are supported by the strong suppressive effect of SMB and CM on the nutrient-consuming microorganisms, most notably yeasts that grow on soluble substrates in the presence of oxygen [29]. Knicky and Spörndly [10] reported that the CM application to various forage crops resulted in a considerable restriction of yeast growth during silage fermentation, with silages remaining aerobically stable throughout the experimental period.

Overall, based on the affordability of the preservative since SMB has a lower price than CM as well as the minimization of the SMB load (less toxicity), the equal combination of SMB and CM was selected as the optimal preservative combination for further experimentation in TMR silage fermentation. The basal TMR used in this experiment was similar to that in Exp. 1. The ingredient and chemical composition of TMR is presented in Supplementary Table S3. Again, with the increased substitution of FVD treated with 2 g SMB + 2 g CM with wet brewers’ grain, WSC and NFC contents increased slightly.

**Figure 5:**
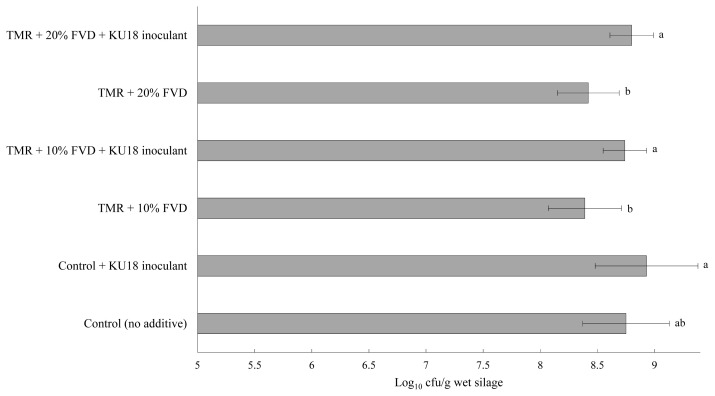
Viable LAB count of TMR silage after 14 days of fermentation with different levels of FVD. FVD were mixed with 2 g chemical mixture + 2 g sodium metabisulfite/kg fresh biomass, and aerobically exposed for 7 days at an outdoor environment. Treatments were TMR containing (as-fed basis) 0%, 10%, or 20% of treated FVD with or without KU18 inoculant. The inoculant was applied uniformly at the rate of 5 mL/kg fresh mass. The chemical mixture was based on sodium benzoate (57%), potassium sorbate (29%), and sodium nitrite (14%). Error bars at each point represent standard error (n = 5). LAB, lactic acid bacteria; TMR, total mixed ration; FVD, fruit and vegetable discards. Bars with different letters differ, based on the Tukey’s test (p-value = 0.04).

**Figure 6:**
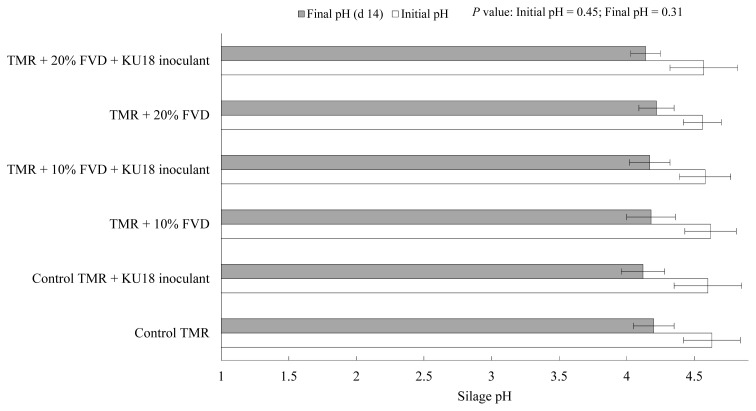
Initial (open bar) and final pH (grey bar; day 14) of TMR silage with different levels of FVD. FVD were mixed with 2 g chemical mixture + 2 g sodium metabisulfite/kg fresh biomass, and aerobically challenged for 7 days at an outdoor environment. Treatments were TMR containing (as-fed basis) 0%, 10%, or 20% of treated FVD with or without KU18 inoculant. The inoculant was applied uniformly at the rate of 5 mL/kg fresh mass (Exp. 2). The chemical mixture was based on sodium benzoate (57%), potassium sorbate (29%), and sodium nitrite (14%). Error bars at each point represent standard error (n = 5). TMR, total mixed ration; FVD, fruit and vegetable discards.

Viable LAB colonies in TMR silage mass after 14 days of fermentation with 0%, 10%, or 20% of FVD, with or without KU18 inoculation, is presented in Figure 5. Compared to the control TMR, a minor inhibitory effect was seen on LAB growth in the TMR silages with 10% or 20% of FVD (treated with 2 g CM + 2 g SMB/kg fresh biomass). The LAB count was higher than 8 log10 cfu/g wet biomass for all silages, with the KU18-inoculated control silage having the highest count of 8.89 log_10_ cfu/g wet biomass. These results are in agreement with the findings presented in Exp. 1 reporting the highest number of LAB in KU18-inoculated control TMR silage. No putrid odor was detected among the experimental silages, and all silages had a desirable fermentative odor with no sign of moldy appearance (data not presented) suggesting that the epiphytic LAB are able to promote efficient silage fermentation. Similar to the results obtained in Exp. 1, a negligible difference in the initial pH among the experimental treatments was observed. However, in contrast to the findings of Exp. 1, a slight variation was observed in silage pH after 14 days of storage (Figure 6), which is supported by the narrow gap in viable LAB count of experimental silages (Figure 5). More data on silage fermentation metabolites such as ammonia as well as the aerobic stability evaluation of the experimental silage are needed to confirm the benefits and efficacy of KU18 inoculation. Such data were not collected in this study as our primary objective was to evaluate the viability of LAB during short-term silage fermentation.

Our previous investigation found that when SMB-treated FVD was co-ensiled with almond hulls and corn gluten feed, free sulfite concentration decreases progressively as a function of the ensiling length. Free sulfite is the active form of SMB providing the strong inhibitory effect against a wide range of microorganisms especially LAB [1]. The suggested mechanism for the decreased content of free sulfite was explained through the formation of bonds between carbonyl constituents in the silage mass and free sulfite [1,30]. Free sulfite also progressively declines when SMB is exposed to oxygen and thus released into the air in the form of SO_2_ [2]. Although we have no experimental data to support this explanation, it appears that the initial free sulfite concentration decreased during the aerobic and initial phase of ensiling periods, which relieves the potential toxic effect of SMB on LAB growth during silage fermentation. More experimentation is required to understand the time course of changes in free sulfite concentration during the aerobic and ensiling storage of FVD treated with the combination of SMB and CM in TMR. The extent of free sulfite reaction with carbonyl constituents in TMR silage mass would reveal more information about the toxic effect of SMB on LAB growth during silage fermentation.

## CONCLUSION

Although among the isolated LAB, KU18 strain was able to grow comparatively more on MRS broth added with SMB, it was not able to effectively dominate on the silage biomass that included 10% or 20% of SMB-treated FVD (6 g SMB/kg fresh FVD mass). As an alternative approach, the minimization of the SMB load with its partial substitution with a CM based on sodium benzoate, potassium sorbate, and sodium nitrite was a successful strategy that enabled the viability and growth of epiphytic LAB during the silage fermentation. An equal combination of SMB and CM (2 g each/kg fresh biomass) was able to effectively preserve the nutrient constituents of FVD during the 7-days aerobic storage. The subsequent inclusion of the SMB + CM-treated FVD into the TMR and their short-term fermentation provided the comparable results in regard to LAB development, silage mass acidification, and fermentative odor to those of control silage.

## Figures and Tables

**Table 1:** Enumeration of the presumptive lactic acid bacteria isolates grown for 12 hours in De Man, Rogosa and Sharpe broth with sodium metabisulfite addition (1,000 mg/L; n = 5)

Presumptive LAB	Viable count, log_10_ cfu/mL
KU1	4.59
KU2	4.65
KU3	5.98
KU4	4.45
KU5	2.30
KU6	4.00
KU7	2.01
KU8	4.33
KU9	4.32
KU10	5.83
KU11	UN^1)^
KU12	2.00
KU13	4.46
KU14	2.60
KU15	UN
KU16	4.46
KU17	UN
KU18	6.10
KU19	5.18
KU20	4.72
KU21	UN
KU22	4.22
KU23	3.41
KU24	2.90
KU25	3.80
KU26	2.93
KU27	4.06
KU28	3.44
KU29	5.71

LAB, lactic acid bacteria; cfu, colony-forming unit.

1) UN, undetected; below the detection limit (2.8 log10 cfu/mL MRS broth).

**Table 2:** Broth culture pH of the selected lactic acid bacteria in MRS broth with sodium metabisulfite addition (1,000 mg/L) as a function of incubation time (n = 5)

LAB strains	Culture time (h)					SE	p-value

	0	3	6	12	24		
KU3	6.31^a^	6.32^a^	6.14^b^	6.04^c^	5.92^d^	0.15	<0.01
KU10	6.33^a^	6.33^a^	6.23^b^	6.15^c^	6.03^d^	0.11	<0.01
KU18	6.33^a^	6.32^a^	6.02^b^	5.94^c^	5.71^d^	0.23	<0.01
KU19	6.32^a^	6.33^a^	6.18^b^	6.05^c^	6.01^c^	0.13	<0.01
KU29	6.34^a^	6.34^a^	6.28^b^	6.13^c^	6.11^c^	0.09	<0.01
*L. plantarum* KU5 (positive control strain)^1)^	6.34^a^	6.36^a^	6.09^b^	5.92^c^	5.83^d^	0.21	<0.01

MRS, De Man, Rogosa and Sharpe; LAB, lactic acid bacteria; SE, standard error.

1) Isolated and identified from spent mushroom substrate with accession No. of HQ542227 (Kim and Kwak [15]).

a–d Means within a row with different superscripts differ (p<0.05).

**Table 3:** Effect of additive treatments on the pH and population of various microbial groups in fruit and vegetable discards after 0 and 7 days of aerobic challenge (n = 5)

Items	Treatments1)2)						SE	p-value

	Control	SMB (6 g)	CM (3 g)	SMB (2 g) + CM (2 g)	SMB (3 g) + CM (1.5 g)	SMB (4 g) + CM (1 g)		
day 0								
pH	4.56^bA^	4.10^aA^	4.72^bB^	4.36^aA^	4.30^aA^	4.27^aA^	0.12	0.02
Total bacteria	7.11^cA^	4.12^bA^	5.96^aB^	5.85^aB^	5.66^aB^	5.79^aA^	0.59	<0.01
LAB	6.05^aA^	<2.8^bA^	5.72^aB^	5.57^aB^	5.12^aB^	4.98^aB^	0.41	<0.01
Yeast	5.08^bA^	<2.8^aA^	<2.8^aA^	4.90^bB^	<2.08^aA^	4.90^bB^	0.30	<0.01
Mold	4.90^bA^	<2.8^aA^	<2.8^aA^	4.60^bB^	5.08^bB^	4.60^bB^	0.28	<0.01
day 7								
pH	3.63^bB^	4.05^aA^	4.11^aA^	4.33^aA^	4.06^aB^	4.22^aA^	0.11	<0.01
Total bacteria	8.25^bB^	4.24^aA^	4.80^aA^	4.42^aA^	4.74^aA^	5.05^aA^	0.48	<0.01
LAB	7.26^aB^	<2.8^cA^	3.15^bA^	<2.8^cA^	<2.8^cA^	<2.8^cA^	0.14	<0.01
Yeast	6.11^bB^	<2.8^aA^	<2.8^aA^	<2.8^aA^	<2.8^aA^	<2.8^aA^	0.11	<0.01
Mold	3.95^bB^	<2.8^aA^	<2.8^aA^	<2.8^aA^	<2.8^aA^	<2.8^aA^	0.01	<0.01

SMB, sodium metabisulfite; CM, chemical mixture; SE, standard error; LAB, lactic acid bacteria.

1) Chemical additive loads are based on g additive/kg of fresh biomass.

2) CM based on sodium benzoate (57%), potassium sorbate (29%), and sodium nitrite (14%).

Microbial counts are logarithm number of colony-forming units per g fresh matter. The limit of detection was 2.8 log cfu per g fresh matter.

a–c Means within each row with different superscript differ (p<0.05).

A,B Means within the same treatment between two periods with different superscript differ (p<0.05).
