# Supplementary material for: A novel combination of sodium metabisulfite and a chemical mixture based on sodium benzoate, potassium sorbate, and sodium nitrite for aerobic preservation of fruit and vegetable discards and lactic acid fermentation in a total mixed ration for ruminants
Source: Anim Biosci. 2021 Mar 2;34(9):1479–90. doi: 10.5713/ab.20.0871 (PMC8495328; doi:10.5713/ab.20.0871)
Supplement: Supplementary file 1 [file ab-20-0871-suppl.pdf]

Supplementary Table S1. Individual ingredient proportion (%) and chemical composition of the total fruit and vegetable discards used in experiments

Individual ingredient[1]	Exp. 1	Exp. 2
Apple	11	18
Grape	15	14
Plum	12	11
Garlic	12	4
Orange	9	10
Onion	10	4
Potato	6	9
Sweet potato	9	5
Melon	8	8
Tomato	0	8
Green onion	3	6
Mushroom	5	—
Paprika	—	3
Nutrient composition (n = 3)		
DM, %	13.8	15.1
Neutral detergent fiber, % of DM	14.2	12.6
Crude ash, % of DM	4.61	4.31
Lignin, % of DM	2.70	2.83
Ether extract, % of DM	1.82	1.53
Crude protein, % of DM	7.48	5.66
NFC, % of DM	71.8	75.9
Water-soluble carbohydrates, % of DM	47.4	48.8

NFC = non-fibrous carbohydrates calculated as 100 – [NDF + crude protein + crude ash + ether extract].

[1] From the input and output data in the packing house, the discarded amount of main individual ingredients during each month that constituted more than 90% of total discards were surveyed and thus collected for the respective experiment.

Supplementary Table S2. Description of TMR in Exp. 1

Ingredients, as-fed basis	Treatment		
	T0	T10	T20
Barely brewers grain, wet	33.0	23.0	13.0
Barley straw bale	13.0	13.0	13.0
Ryegrass hay	13.0	13.0	13.0
Alfalfa hay	10.0	10.0	10.0
Corn grain, cracked	18.0	18.0	18.0
Fruit and vegetable discards*	0.0	10.0	20.0
Soybean meal	6.0	6.0	6.0
Beet pulp, dried	5.60	5.60	5.60
Salt	0.20	0.20	0.20
Limestone	0.60	0.60	0.60
Vitamin and mineral premix	0.60	0.60	0.60
Nutrient composition (n = 3)			
Dry matter (DM), %	64.5	63.6	62.6
Crude protein, % of DM	13.5	12.9	12.4
Ether extract, % of DM	2.68	2.54	2.40
Crude ash, % of DM	6.25	6.34	6.44
Neutral-detergent fiber, % of DM	37.3	36.9	36.4
Non-fibrous carbohydrates, % of DM	40.3	41.3	42.4
Water-soluble carbohydrates, % of DM	4.67	6.22	7.82
Calcium, % of DM	0.68	0.69	0.69
Phosphorus, % of DM	0.36	0.35	0.34

* Fruit and vegetable discards (FVD) were mixed with 6 g sodium metabisulfite/kg fresh biomass and aerobically stored for 7 days at an outdoor environment.
The treated FVD were included in TMR (as-fed basis) at 0, 10, or 20% level.

Supplementary Table S3. Description of TMR in Exp. 2

Ingredients, as-fed basis	Treatment		
	T0	T10	T20
Barely brewers grain, wet	33.0	23.0	13.0
Barley straw bale	13.0	13.0	13.0
Ryegrass hay	13.0	13.0	13.0
Alfalfa hay	10.0	10.0	10.0
Corn grain, cracked	18.0	18.0	18.0
Fruit and vegetable discards*	0.0	10.0	20.0
Soybean meal	6.0	6.0	6.0
Beet pulp, dried	5.60	5.60	5.60
Salt	0.20	0.20	0.20
Limestone	0.60	0.60	0.60
Vitamin and mineral premix	0.60	0.60	0.60
Chemical composition (n = 3)			
Dry matter (DM), %	63.5	63.1	62.6
Crude protein, % of DM	13.9	13.3	12.9
Ether extract, % of DM	2.43	2.47	2.37
Crude ash, % of DM	6.11	6.45	6.52
Neutral-detergent fiber, % of DM	36.1	34.8	35.1
Non-fibrous carbohydrates, % of DM	41.4	43.0	43.1
Water-soluble carbohydrates, % of DM	4.71	5.98	6.87
Calcium, % of DM	0.68	0.67	0.68
Phosphorus, % of DM	0.34	0.37	0.36

* Fruit and vegetable discards (FVD) were mixed with 2 g chemical mixture + 2 g sodium metabisulfite/kg fresh biomass and aerobically stored for 7 days at an outdoor environment.

Chemical mixture was based on sodium benzoate (57%), potassium sorbate (29%), and sodium nitrite (14%).

The treated FVD were included in TMR (as-fed basis) at 0, 10, or 20% level.

Supplementary Fig. 1. 16S rRNA sequence of KU18 strain (Exp. 1)

CATTTGAGTGAGTGGCGAACTGGTGAGTAACACGTGGGAAACCTGCCCAG
AAGCGGGGGATAACACCTGGAAACAGATGCTAATACCGCATAACAACTTG
GACCGCATGGTCCAAGTTTGAAAGATGGCTTCGGCTATCACTTTTGGATG
GTCCCGCGGCGTATTAGCTAGATGGTGGGGTAACGGCTCACCATGGCAAT
GATACGTAGCCGACCTGAGAGGGTAATCGGCCACATTGGGACTGAGACAC
GGCCCAAACTCCTACGGGAGGCAGCAGTAGGGAATCTTCCACAATGGACG
AAAGTCTGATGGAGCAACGCCGCGTGAGTGAAGAAGGGTTTCGGCTCGTA
AAACTCTGTTGTTAAAGAAGAACATATCTGAGAGTAACTGTTCAGGTATT
GACGGTATTTAACCAGAAAGCCACGGCTAACTACGTGCCAGCAGCCGCGG
TAATACGTAGGTGGCAAGCGTTGTCCGGATTTATTGGGCGTAAAGCGAGC
GCAGGCGGTTTTTTAAGTCTGATGTGAAAGCCTTCGGCTCAACCGAAGAA
GTGCATCGGAAACTGGGAAACTTGAGTGCAGAAGAGGACAGTGGAACTCC
ATGTGTAGCGGTGAAATGCGTAGATATATGGAAGAACACCAGTGGCGAAG
GCGGCTGTCTGGTCTGTAACTGACGCTGAGGCTCGAAAGTATGGGTAGCA
AACAGGATTAGATACCCTGGTAGTCCATACCGTAAACGATGAATGCTAAG
TGTTGGAGGGTTTCCGCCCTTCAGTGCTGCAGCTAACGCATTAAGCATTC
CGCCTGGGGAGTACGGCCGCAAGGCTGAAACTCAAAGGAATTGACGGGGG
CCCGCACAAGCGGTGGAGCATGTGGTTTAATTCGAAGCTACGCGAAGAAC
CTTACCAGGTCTTGACATACTATGCAAATCTAAGAGATTAGACGTTCCCT
TCGGGGACATGGATACAGGTGGTGCATGGTTGTCGTCAGCTCGTGTCGTG
AGATGTTGGGTTAAGTCCCGCAACGAGCGCAACCCTTATTATCAGTTGCC
AGCATTAAGTTGGGCACTCTGGTGAGACTGCCGGTGACAAACCGGAGGAA
GGTGGGGATGACGTCAAATCATCATGCCCCTTATGACCTGGGCTACACAC
GTGCTACAATGGATGGTACAACGAGTTGCGAACTCGCGAGAGTAAGCTAA
TCTCTTAAAGCCATTCTCAGTTCGGATTGTAGGCTGCAACTCGCCTACAT
GAAGTCGGAATCGCTAGTAATCGCGGATCAGCATGCCGCGGTGAATACGT
TCCCGGGCCTTGTACACACCGCCCGTCACACCATGAGAGTTTGTAACACC
CAAAGTCGGTGGGGTAACCTTTTAGGAACCAGCCGCCTAAGGTGGGACAG
ATGATTAGGGTGAAGTCGTAACAAGGTAGCCGTAGGAGAACCTGCGGCTG
